# Therapeutic Potential and Recent Advances of Curcumin in the Treatment of Aging-Associated Diseases

**DOI:** 10.3390/molecules23040835

**Published:** 2018-04-05

**Authors:** Sathish Sundar Dhilip Kumar, Nicolette Nadene Houreld, Heidi Abrahamse

**Affiliations:** Laser Research Centre, Faculty of Health Sciences, University of Johannesburg, Johannesburg-2028, South Africa; sathishd@uj.ac.za (S.S.D.K.); nhoureld@uj.ac.za (N.N.H.)

**Keywords:** curcumin, nanoparticle, plants, natural products, drug delivery, aging

## Abstract

Curcumin, a low molecular weight, lipophilic, major yellow natural polyphenolic, and the most well-known plant-derived compound, is extracted from the rhizomes of the turmeric (*Curcuma longa*) plant. Curcumin has been demonstrated as an effective therapeutic agent in traditional medicine for the treatment and prevention of different diseases. It has also shown a wide range of biological and pharmacological effects in drug delivery, and has actively been used for the treatment of aging-associated diseases, including cardiovascular diseases, atherosclerosis, neurodegenerative diseases, cancer, rheumatoid arthritis, ocular diseases, osteoporosis, diabetes, hypertension, chronic kidney diseases, chronic inflammation and infection. The functional application and therapeutic potential of curcumin in the treatment of aging-associated diseases is well documented in the literature. This review article focuses mainly on the potential role of plant-derived natural compounds such as curcumin, their mechanism of action and recent advances in the treatment of aging-associated diseases. Moreover, the review briefly recaps on the recent progress made in the preparation of nanocurcumins and their therapeutic potential in clinical research for the treatment of aging-associated diseases.

## 1. Introduction

Aging is an inevitable process for all organisms. It creates numerous cellular and molecular malfunctions which lead to the development of various chronic diseases, also generally referred to as aging-associated diseases. The occurrence of aging-associated diseases is increasing worldwide and becoming the leading causes of disability and increased risk of mortality [1,2,3]. Some of the most common aging-associated diseases are atherosclerosis, cancer, cardiovascular diseases, chronic inflammation, chronic kidney diseases, diabetes, neurodegenerative diseases, hypertension, rheumatoid arthritis, ocular diseases, osteoporosis, and infections [4,5]. Aging-associated diseases usually develop in middle age after the prolonged exposure to an unhealthy early-life environment involving the excessive usage of tobacco and alcohol, anxiety, lack of physical activity and the consumption of a high-fat diet [1]. Clinical trials and epidemiological data has suggested that healthy lifestyle interventions help to prevent many aging-associated chronic diseases, and many can even be reversed [6].

Plant-derived natural products play a vital role in the prevention and treatment of aging-associated diseases [7]. Natural products extracted from plants has been used as an active ingredient in traditional medicine for many centuries [8]. Some plant-derived natural products possess a wide range of biological and pharmacological properties that have become indispensable in modern pharmacotherapy, e.g., paclitaxel (an anticancer drug) from *Taxus brevifolia*, vincristine and vinblastine (anticancer drugs) from *Catharanthus roseus,* camptothecin (anticancer drug) from *Camptotheca acuminata* and quercetin (anticancer drug), a polyphenol found in various vegetables and fruits [9,10,11]. Curcumin, which has the chemical structure 1,7-bis(4-hydroxy-3-methoxyphenyl)-1,6-heptadiene-3,5-dione, is a natural yellow, polyphenolic pigment compound extracted from the rhizomes of turmeric (*Curcuma longa*), is among these plant-derived natural products [12,13]. It is used as a food ingredient in all forms of Indian cuisine, and it shows a wide range of pharmacological activities. It is used for the treatment of neurodegenerative [14], atherosclerosis [15], cardiovascular [16], cancer [17], pulmonary, autoimmune and neoplastic diseases [18], biliary and hepatic disorders [19], diabetes [20], wound healing [21], rheumatoid arthritis [22], chronic inflammation [23], chronic kidney diseases [24], hypertension [25], ocular diseases [26], osteoporosis [27], and skin diseases [28]. Although curcumin possesses good therapeutic efficacy in the treatment of different diseases, the clinical administration of curcumin is difficult due to its poor oral bioavailability, low solubility in aqueous solution, and degradation under physiological conditions [13]. 

Nanocarrier-mediated delivery aids in overcoming the problems associated with curcumin and facilitates successful delivery without affecting its therapeutic efficacy. Several carrier molecules are used to enhance the bioavailability of curcumin such as polymeric nanoparticles (natural and synthetic polymers), liposomes, dendrimers, solid lipid nanoparticles, and gold nanoparticles [29]. Recent research has suggested that nanoformulated curcumin has a better therapeutic index than the native form of curcumin [30]. In this review article we primarily emphasize the therapeutic potential of curcumin and the possible advantages of using nanocurcumin, which possesses higher stability and increased bioavailability than free curcumin, in the prevention and treatment of aging-associated diseases. 

## 2. Curcumin

**Figure 1:**
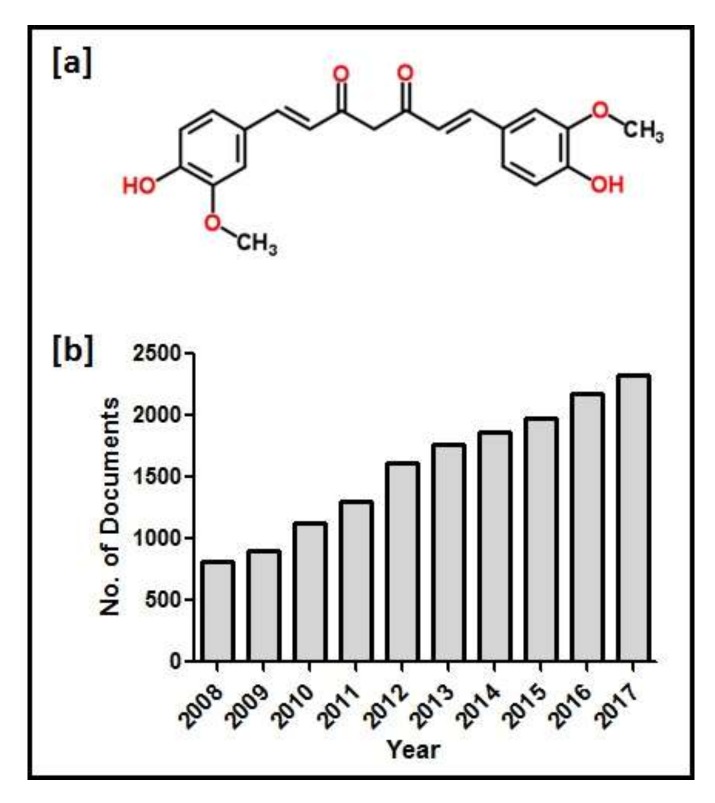
(**a**) Chemical structure of curcumin; and (**b**) annual publication history of curcumin (2008–2017).

Turmeric, the golden spice also known as “Indian saffron” is used as a traditional medicine in Southeast Asia [31]. The most important bioactive chemical constituents of turmeric are curcuminoids, which include curcumin, demethoxycurcumin and bisdemethoxycurcumin [32]. Curcumin, a hydrophobic polyphenol, is extracted from the rhizome of the herb *Curcuma longa*, which belongs to the *Zingiberaceae* family [33]. It has a wide spectrum of biological and pharmacological activities [34]. Curcumin is less soluble in water and highly soluble in organic solvents including methanol, ethanol, acetone and dimethyl sulfoxide [35]. Curcumin is commonly called diferuloylmethane, and it gives a yellow color to turmeric powder. The chemical structure is shown in Figure 1a. The ultraviolet-visible surface plasmon resonance spectrum of curcumin exhibits a strong absorption peak between 420–430 nm. In the last 10 years, over 15,000 scientific papers have been published on curcumin, and these numbers have been increasing annually. Scopus results for the period 2008–2017 are shown in Figure 1b. 

**Figure 2:**
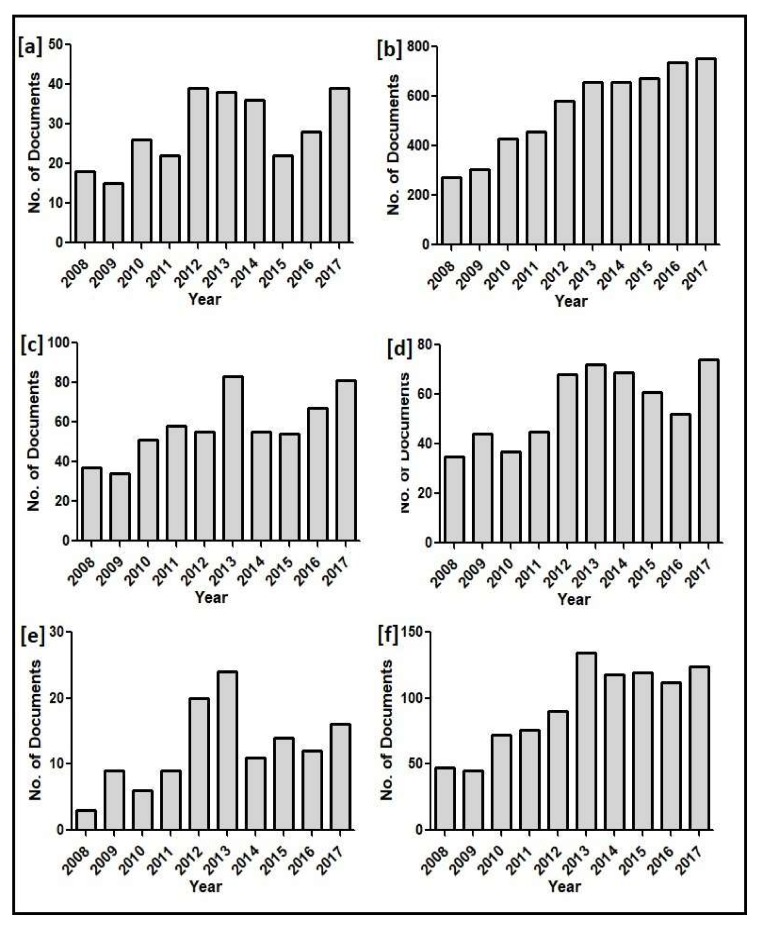

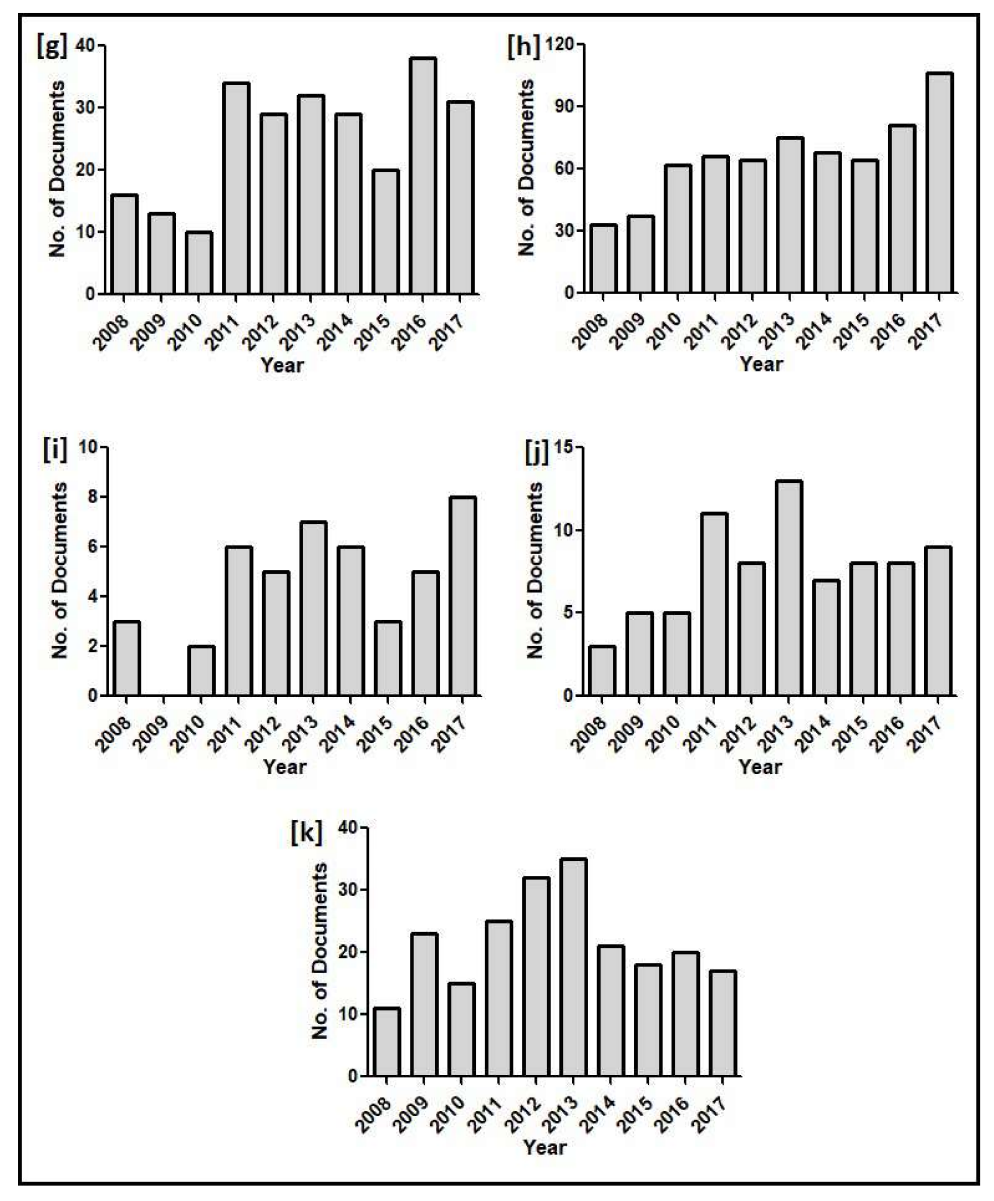
Annual publication history (2008–2017) of curcumin with different aging-associated diseases generated from the “Scopus” database, using the keywords (**a**) atherosclerosis-curcumin, (**b**) cancer-curcumin; (**c**) cardiovascular diseases-curcumin (**d**) chronic inflammation-curcumin; (**e**) chronic kidney diseases-curcumin; (**f**) diabetes-curcumin; (**g**) hypertension-curcumin; (**h**) neurodegenerative diseases-curcumin; (**i**) ocular diseases-curcumin; (**j**) osteoporosis-curcumin; and (**k**) rheumatoid arthritis-curcumin.

An extensive literature survey was conducted over the last 10 years on aging-associated diseases and their treatment strategies using the drug curcumin. Atherosclerosis-curcumin, cancer-curcumin, cardiovascular diseases-curcumin, chronic inflammation-curcumin, chronic kidney diseases-curcumin, diabetes-curcumin, hypertension-curcumin, neurodegenerative diseases-curcumin, ocular diseases-curcumin, osteoporosis-curcumin, and rheumatoid arthritis-curcumin were used as keywords to search the “Scopus” database for the period 2008–2017, and the results are represented in Figure 2. The literature study describes the annual publication history and the recent research progress of curcumin in relation to the above-mentioned aging-associated diseases.

### Biological and Pharmacological Properties of Curcumin

Currently, curcumin is being investigated as an active therapeutic agent for several types of diseases including cardiovascular, pulmonary, skin disorders, liver disorders, fatigue, neuropathic pain, bone and muscle loss, and anxiety, due to its well reported biological activities which include anti-inflammatory, anti-proliferative, anti-angiogenic, pro-apoptotic, anti-oxidant, wound healing, anti-cancer, anti-viral, and anti-diabetic effects [36,37,38]. It is a well-recognized plant-based drug for the treatment of most chronic diseases. Curcumin has exhibited excellent anti-bacterial activity against both methicillin resistant and sensitive *Staphylococcus aureus* [39]. It is also one of the most frequently prescribed drugs for the treatment of different skin diseases and skin cancer [28]. 

Extensive investigation has indicated that curcumin resulted in a reduction in total cholesterol and low-density lipoprotein (LDL) levels in patients suffering from acute coronary syndrome [40], and that it effectively reduced platelet aggregation and hyperlipidemia [41]. Curcumin possesses anti-thrombotic activities and daily consumption helps to maintain anticoagulant status [42]. It can promote cardiac repair and restore cardiac dysfunction [43], molecular understanding in diabetic nephropathy [44], modulate cell cycle regulatory proteins in central nervous system-related disorders including multiple sclerosis [45], and it can be potentially used for the treatment of hepatic diseases [46], and different types of cancers [47]. The beneficial effects of curcumin have also been studied in detail in ocular diseases, which include corneal diseases, eye dryness, conjunctivitis, anterior uveitis, pterygium, regulating calcium hemostasis, cataracts and glaucoma [48].

Various *in vitro* and *in vivo* preclinical studies have demonstrated that curcumin has effective biological pharmacological and potential clinical applications by interacting with numerous cellular and molecular targets via a diverse range of mechanisms of action [49], which are presented in Table 1. 

## 3. Therapeutic Potential of Curcumin in Aging-Associated Diseases

The aging process is related to a wide range of disorders. Aging-associated diseases are threatening the elderly population worldwide. Research over the last few decades has studied the therapeutic potential of curcumin and showed the beneficial aspects of curcumin in the prevention and treatment of aging-associated diseases. The therapeutic potential of curcumin used in the treatment of aging-associated diseases is discussed in Table 2.

## 4. Recent Advances of Nanocurcumin in Aging-Associated Diseases

In recent years, nanotechnology-based drug delivery systems have received considerable attention in drug delivery and pharmaceutical research due to their capability to deliver drugs and other substances (such as targeting ligands, fluorescent dyes) to the targeted region. Nanoformulation offers distinct advantages in drug delivery which include lack of toxicity, high drug loading capacity, controlled drug release, extended shelf life period, higher stability, functionalization with specific targets, intracellular release and targeted delivery [62]. Various nano-based formulations are successfully studied for imaging, diagnostic and therapeutic applications [63]. Recent advances in nanotechnology have facilitated the formulation of nanocurcumin and it significantly improved the therapeutic efficacy and bioavailability of its native counterpart [64] and provides the opportunity for disease specific targeting [65]. 

Curcumin-loaded spherical polymeric nanoconstructs with lipid chains was assessed *in vitro* using RAW 264.7 cells. The nanoconstructs significantly modulated the production of pro-inflammatory cytokines without any cytotoxic effect [66]. Curcumin-conjugated magnetic nanoparticles bind with amyloid plaques in mouse brains, which was confirmed through immunohistochemical studies and magnetic resonance images. This study revealed the potential application of curcumin magnetic nanoparticles to visualize amyloid plaques in Alzheimer’s disease [67]. Curcumin loaded zinc oxide nanoparticles encapsulated with a co-polymer were successfully synthesized, and their anticancer activity showed excellent cytotoxicity effects in AGS gastric cancer cells [68]. *In vitro* and *in vivo* studies showed that curcumin encapsulated PLGA nanoparticles showed effective targeting and growth inhibition of prostate cancer cells [69]. Meena et al. synthesized small positively charged curcumin nanoparticles using PLGA as a carrier, and tested these nanoparticles in triple-negative breast cancer cells. Nanocurcumin showed higher cellular incorporation and produced more cytotoxic effects than free curcumin, and further apoptosis induction occurred due to DNA damage [70]. Fan et al. fabricated curcumin loaded nanoparticles using bovine serum albumin and dextran as a carrier molecule, and showed improved cellular antioxidant activity in Caco-2 cells [71]. Curcumin loaded solid lipid nanoparticles were synthesized and were revealed as a safe and effective therapeutic agent for the treatment of lipopolysaccharide-induced sepsis in IL-1b transgenic mice [72]. Curcumin nanoparticles possessed cardio protection against palmitate induced cardiomyocyte apoptosis in H9C2 embryonic rat heart-derived cells, and may be an alternative treatment for lipid toxicity and myocardial injury [73]. Dewangan et al. prepared curcumin-carboxymethyl cellulose acetate butyrate nanoparticles in the size range of 166.5 ± 4.2 nm with higher loading efficiency. This study demonstrated superior anti-inflammatory effects without any specific side effects for the successful treatment on adjuvant induced arthritis in rats [74]. The curcumin encapsulated PLGA nanoparticle approach enhanced the effect of curcumin against bone loss. It showed significant beneficial effects on osteoporotic bone loss treatment with an improved therapeutic index [75]. Curcumin encapsulation was performed using a novel nanomicelle formulation which significantly improved *in vitro* cellular uptake and *in vivo* corneal permeation as compared to free curcumin [76]. Some to the recent advances in the preparation of nanocurcumin for the treatment of aging-associated diseases are well studied and are listed in Table 3.

## 5. Clinical Application of Curcumin in Aging-Associated Diseases

**C**urcumin has been extensively used in clinical trials and is showing positive outcomes for the treatment of aging-associated diseases. The global market is predominantly focused on curcumin as a health supplement due to its effective antioxidant and anti-inflammatory properties [39]. The different aging-associated diseases along with curcumin were used as a keyword to search the “https://clinicaltrials.gov” database. The number of clinical trials are listed in Table 4 and some of the major global clinical trial status of curcumin in aging-associated diseases are listed in Table 5. 

## 6. Future Perspectives and Conclusions 

In this review, we discussed in some detail the therapeutic potential of curcumin for the prevention and treatment of aging-associated diseases. Curcumin is a well-known plant-derived compound that exhibits a wide range of biological and pharmacological effects in modern medicine. It is well documented in the literature and shows positive effects on the treatment of aging-associated diseases. However, despite the fact that the native form of curcumin possesses good therapeutic efficacy, the clinical administration is problematic due to their poor oral bioavailability and stability. Recent advances in nanotechnology have helped to overcome these difficulties and efficiently deliver curcumin to the targeted region thereby improving curcumin bioavailability. An extensive literature study strongly suggests that the nanocarrier-mediated delivery of curcumin will become a successful treatment strategy for aging-associated diseases. Moreover, future perspectives should include additional clinical trial investigations on the formulation of nanocurcumin with improved stability and oral bioavailability, and focus more on targeted drug delivery of curcumin for the treatment of aging-associated diseases.

## Figures and Tables

**Table 1:** Diverse range of mechanisms of action of curcumin in aging-associated diseases.

Aging-Associated Diseases	Mechanism of Action	References
Atherosclerosis	Reduces cholesterol accumulation; downregulates SR-A and upregulates ABCA1 by a proteasome and LXRα dependent pathway; inhibition of low density lipoprotein oxidation; stabilization of cell membrane cholesterol.	[50,51,52]
Cancer	Induction of apoptotic signal; suppression of anti-apoptotic proteins; modulates miRNAs, Wnt/β-Catenin signals, protein kinases, and proteasome activation; inhibition of NF-κB; reverses the multidrug resistance of cancer cells.	[53]
Cardiovascular diseases	Anti-oxidant (inhibits eNOS, and iNOS); inhibits sarcoplasmic Ca^2+^-ATPase; membrane stabilizing effect; prevents Adriamycin-induced cardiomyopathy; prevents diabetic cardiovascular complications; inhibits p300 and NF-κB; decreases serum cholesterol levels.	[16]
Chronic inflammation	Alleviates oxidative stress; suppress pro-inflammatory pathways; TNF blocker.	[23]
Chronic kidney diseases	Reduces inflammatory molecules MCP-1, NF-κB, TNF-α, IL-1β, COX-2 and cav-1; induces the expression of anti-inflammatory factors such as HO-1, M6PRBP1, and NEDD4.	[54]
Diabetes	Reduces oxidative stress; reduction of blood glucose levels; stimulates insulin production.	[55]
Hypertension	Reduces AT_1_R mediated vasoconstriction.	[25]
Neurodegenerative diseases	Lowers cholesterol levels; inhibits amyloid-β; modulates microglia; Acetylcholinesterase and Tau inhibition; copper binding; anti-oxidant properties.	[56]
Ocular diseases	Neuroprotection in glaucoma; anti-inflammation activities in anterior uveitis and dry eye; anti-allergy in conjunctivitis; anti-proliferation activities and promotes apoptosis in pterygium; inhibits neovascularization; anti-oxidative stress in cataracts; protects epithelial barriers and promotes wound healing in corneal diseases.	[48]
Osteoporosis	Suppresses the expression of MMP-9; protective effect against bone deterioration.	[57]
Rheumatoid arthritis	Anti-inflammatory activity; inhibits enzymes involved in inflammation (COX-2); downregulates the activation of transcription factor NF-κB and expression of other inflammatory intermediate molecules (TNF-α, adhesion molecules, MMPs, COX-2, and 5-LOX) associated with arthritis.	[58]

ABCA1—ATP binding cassette family member 1; LXRα-Liver X receptor alpha; miRNAs—microRNAs; eNOS—endothelial nitric oxide synthase; NF-κB-Nuclear factor kappa B; iNOS—Inducible nitric oxide synthase; TNF—Tumor necrosis factor; MCP-1-Monocyte Chemoattractant Protein-1; IL-1β—Interleukin 1 beta; COX-2 —Cyclooxygenase 2; cav-1—Caveolin 1; HO-1—Heme oxygenase 1; M6PRBP1-Mannose-6-phosphate receptor binding protein 1; NEDD4-neural precursor cell expressed developmentally down-regulated protein 4; AT1R-Angiotensin II receptor type 1; MMP-9—Matrix metallopeptidase 9; MMPs—Matrix metalloproteinase; 5-LOX—5 lipoxygenase.

**Table 2:** Therapeutic potential of curcumin used in the treatment of aging-associated diseases.

Aging-Associated Diseases	Therapeutic Potential of Curcumin	Outcomes	References
Atherosclerosis	Anti-atherogenic effect of curcumin via different mechanisms.	The effect of curcumin was studied and compared with the drug lovastatin, and the long-term treatment with curcumin lowered plasma and hepatic cholesterol, and suppressed early atherosclerotic lesions.	[59]
Cancer	Curcumin interferes with multiple cell signaling pathways which include cell cycle, apoptosis, proliferation, angiogenesis, invasion, metastasis and inflammation.	The anticancer activity of curcumin was reported against different cancers, and it plays a significant role in different cell signaling pathway and numerous molecular targets. The *in vitro, in vivo* and clinical studies revealed the therapeutic value of curcumin for the treatment of “old-age“ diseases like cancer.	[60]
Cardiovascular diseases	The inflammatory effects of curcumin may play a key role in the prevention of cardiovascular diseases.	Curcumin showed the possibility of preventing atrial arrhythmias and some ventricular arrhythmias.	[16]
Chronic inflammation	Curcumin can suppress both acute and chronic inflammation by scavenging reactive oxygen species and enhancing antioxidant defences.	Curcumin shows very good antioxidant properties, and it plays a key role in the prevention and treatment of chronic inflammation.	[23,61]
Chronic kidney diseases	Curcumin increases the expression of intestinal alkaline phosphatase and tight junction proteins.	The study shows the potential anti-inflammatory effects of curcumin and their positive effects for the treatment of chronic kidney diseases.	[24]
Diabetes	Curcumin reduces glycemia and hyperlipidemia in rodent models, and favourably affects some leading aspects of diabetes which include insulin resistance, hyperglycemia and islet apoptosis and necrosis.	Curcumin is actively involved in the prevention and treatment of diabetes. The study showed that curcumin and their complexes can successfully be used for the treatment of various disorders associated with diabetes such as liver disorders, adipose tissue dysfunction, diabetic neuropathy, diabetic nephropathy, diabetic vascular disease, and other complications associated with diabetes.	[20]
Hypertension	Curcumin shows beneficial effects on hypertension; it prevents the development of hypertension by regulating AT_1_ receptor expression.	The study was performed using curcumin in an Ang II-induced hypertensive model, and it showed that curcumin aids in the downregulation of the AT_1_ receptor in A10 cells and it subsequently prevents hypertension.	[25]
Neurodegenerative diseases	Curcumin has reported to be an effective neuroprotective agent and it may prevent aging associated changes in cellular proteins.	Protein homeostasis plays an important role in aging-associated diseases. The study performed in an invertebrate model (*Caenorhabditis elegans*) showed that the drug curcumin aids to maintain protein homeostasis and increases the life span of the model organism. Several animal model studies showed that curcumin prevents or delays various neurodegenerative diseases.	[14]
Ocular diseases	Curcumin exhibits potential therapeutic activity against several ocular diseases.	Curcumin showed beneficial effects in the prevention and treatment of several ocular diseases. The dosage of curcumin, up to 8g/day for three months, does not produce any dose-limiting toxicity in pharmacological studies. Clinical data proved that the few weeks of curcumin treatment reduced the signs and symptoms of eye discomfort and is safe in the treatment of humans.	[26]
Osteoporosis	Curcumin may be a potential candidate for the treatment of osteoporosis.	The protective effects of curcumin were studied against dexamethasone induced osteoporosis in a rat model. The results proved that curcumin effectively prevented glucocorticoid- induced osteoporosis.	[27]
Rheumatoid arthritis	Curcumin possess various pharmacological activities including antiarthritic effects.	The effect of curcumin was studied in an adjuvant-induced arthritis rat model, and it showed similar therapeutic effects for the treatment of rheumatoid arthritis compared with the drug methotrexate.	[22]

**Table 3:** Recent advances in the preparation of nanocurcumin for the treatment of aging-associated diseases.

Aging-Associated Diseases	Detail of Nanoparticles	Size (nm)	Outcomes	References
Cancer	Curcumin loaded cationic liposome- polyethylene glycol (PEG) and poly (ethylene imine) complex	270 nm	Curcumin liposomes showed five-fold cytotoxic activity on curcumin-sensitive cells and twenty-fold against curcumin-resistant cells compared to native curcumin, and achieved 45 ± 0.2% curcumin encapsulation efficiency in the liposome complex. *In vivo* studies showed that the administration of curcumin liposomes inhibited 60–90% of tumor growth.	[77]
Cancer	Curcumin loaded silk fibroin nanoparticles	155–170 nm	Curcumin loaded silk fibroin nanoparticles were synthesized using both physical adsorption (drug loading content of 6.63 ± 0.09% and encapsulation efficiency of 53.75 ± 0.81%) and co-precipitation (drug loading content of 2.47 ± 0.11% and encapsulation efficiency of 48.84 ± 2.67%). The synthesized material showed excellent antitumor activity against both Hep3B and Kelly cells.	[78]
Cardiovascular diseases	Colloidal nanoparticles (Curcumin with gum ghatti solution)	190 nm	A small amount of synthesized colloidal curcumin nanoparticles could be more therapeutically effective for heart failure than native curcumin.	[79]
Chronic inflammation	Nano-emulsified curcumin (NEC)	-	NEC was orally supplied to NZM2410 mice (lupus nephritis model) and kidney function was monitored by testing blood urea nitrogen. Results suggested that NEC has a good therapeutic potential in the treatment of chronic inflammation and other autoimmune diseases.	[80]
Chronic kidney diseases	Curcumin nanoparticles	80–100 nm	The *in vitro* and *in vivo* study suggested that the curcumin nanoparticles enhanced the treatment efficacy of Rhabdomyolysis induced acute kidney injury than free curcumin. The release study was performed using dialysis. The initial 20 h of dialysis showed that upto 40% of curcumin nanoparticles were released and 80% of free curcumin was released. Nanoparticulate curcumin achieved prolonged release profile than free curcumin.	[81]
Neurodegenerative diseases	Nanocurcumin (Curcumin loaded p(PEG-poly-lactic acid) micelles	80 nm	There was improved bioavailability of nanocurcumin in the brains of Tg2576 mice as compared to free curcumin, and nanocurcumin showed positive effects in the treatment of Alzheimer’s disease. The entrapment efficiency of curcumin was almost 100% and the loading efficiency of curcumin was 37.6%.	[82]
Ocular diseases	Curcumin encapsulated PLGA nanoparticles	282.50 ± 5.72 nm	The study demonstrated the potential of curcumin encapsulated nanoparticles in managing diabetic cataracts in a streptozotocin induced diabetic rat cataract model. The enhanced performance of nanocurcumin was observed in different biochemical pathways than free curcumin. It may be due to the improved oral bioavailability of curcumin.	[83]
Osteoporosis	Gold nanoparticles functionalized with cyclodextrin curcumin complexes	36.3 nm	Loading efficiency of curcumin was around 38.95% and the curcumin loaded nanoparticle complex showed an effective intracellular uptake and acts as a potential therapeutic agent in the treatment of bone diseases associated with excessive bone resorption.	[84]
Rheumatoid arthritis	Curcumin nanoemulsion (curcumin, solutol-HS 15, soybean oil)	150 nm	*In vivo* rat study showed that the synthesized curcumin nanoemulsion acts as an effective antiarthritic agent. The oral route of curcumin nanoemulsion showed threefold increase of AUC (area under the curve) and C_max_ value than the suspension (intravenous (iv) route) and it significantly enhanced the drug absorption than free curcumin. Overall, the *in vivo* rat study suggested that the nanoform of curcumin aids to convert the therapy route from iv to oral administration for the effective treatment of RA therapy	[22]

**Table 4:** Number of clinical trials generated from the “https://clinicaltrials.gov” database using the keywords (curcumin and the different aging-associated diseases).

Keywords Used	No. of Studies Found
Atherosclerosis-curcumin	1
Cancer-curcumin	57
Cardiovascular diseases-curcumin	9
Chronic inflammation-curcumin	2
Chronic kidney diseases-curcumin	5
Diabetes-curcumin	11
Hypertension-curcumin	1
Neurodegenerative diseases-curcumin	6
Ocular diseases-curcumin	4
Osteoporosis-curcumin	0
Rheumatoid arthritis-curcumin	2

**Table 5:** Status of some of the major global clinical trials of curcumin in aging-associated diseases.

ClinicalTrials.gov Identifier	Ages (Years)	Disease	Doses of Curcumin	Phase	Reference
NCT02998918	18 to 60 (Adult)	Inflammation Atherosclerosis Cardiovascular Disease	500 mg of curcumin phytosome twice daily for 1 week	2	[85]
NCT00973869	18 and older (Adult, Senior)	Colorectal cancer	Oral curcumin once daily for 14–28 days	1	[86]
NCT02099890	18 and older (Adult, Senior)	Neuropathic pain; Depression; Cognitive impairment; Somatic neuropathy; Autonomic dysfunction	InflanNox capsule (400 mg curcumin) taken 3 times daily along with other anti-inflammatory supplements	3	[87]
NCT02369549	18 and older (Adult, Senior)	Chronic kidney disease	Three 30 mg capsules of micro-particle curcumin daily in the morning	3	[88]
NCT02529969	40 to 65 (Adult)	Non-insulin dependent diabetes	500 mg curcumin capsule	2	[89]
NCT02984813	18 and older (Adult, Senior)	Open-angle glaucoma diabetic retinopathy	Two pills daily (one contains curcumin and other contains active compounds) for 3 months	1	[90]
NCT00164749	50 and older (Adult, Senior)	Alzheimer’s disease	Two different dosages (1 g/day and 4 g/day) along with ginkgo extract	2	[91]
NCT00752154	18 and older (Adult, Senior)	Rheumatoid arthritis	Curcumin (Longvida™) 4 capsules approximately 2 g/day for 2 weeks, then the dose will be increased to 4 capsules twice a day (4 g/day)	Early phase 1	[92]
